# Rupatadine oral solution improves rhino-conjunctive symptoms control in children with 6-11 years weighing ≥25 kg with persistent allergic rhinitis

**DOI:** 10.1186/2045-7022-3-S2-P33

**Published:** 2013-07-16

**Authors:** Iñaki Izquierdo, Paul Potter, Jorge Maspero, Jan Vermeulen, Laszlo Barkai, Ildiko Nemeth, Rene Baillieau, Jesus M Garde M, Josep Giralt, Alejandro Domenech, Antonio Nieto

**Affiliations:** 1 Uriach Pharma, Clinical Research, Palau de Plegamans, Spain; 2 Allergy Diagnostic and Clinical Research Unit, University Cape Town, South Africa; 3 Fundacion CIDEA, Allergy Department, Buenos Aires, Argentina; 4 Parow Research, Allergic Dept, Cape Town, South Africa; 5 BAZ Megyei Korhaz, Gyermekegeszsegugyl Kozpont, Miskolc, Hungary; 6 Szent Erzsebet Korhaz, Non-profit Kozhasznu Kft, Jaszbereny, Hungary; 7 Centro de Alergia, Centro de Alergia, Mar de Plata, Argentina; 8 Servicio de Alergia, Hosp G. Universitario de Elche, Elche, Spain; 9 Pediatric Allergy and Pneumology Unit, Children's Hosp La Fe, Valencia, Spain

## Background

Clinical trials with the newer 2nd generation antihistamines in children under the age of 12 years have been performed previously but further studies are needed in order to show efficacy and safety in the most unfavourable clinical conditions such as persistent allergic rhinitis (PER). Rupatadine oral solution was developed for children with allergic rhinitis in view of its rapid onset of action and its lack of relevant side effects. These advantages were confirmed previously in a phase III study in children 6-11 years.

## Objective

To assess the efficacy and safety of rupatadine (RUP) oral solution in a subgroup of children between 6 and 11 years weighing ≥25 kg with PER.

## Methods

A subanalysis was performed from a previous placebo-controlled study carried out in patients between 6-11 years diagnosed as PER according to ARIA criteria. This analysis included patients with a positive prick test, weight ≥25 kg and basal nasal symptoms score (including rhinorrhea, nasal blockage, sneezing and nasal itching assessment) ≥24 obtained in 4 days throughout the 2-week screening period. Patients were allocated to treatment with either RUP oral solution (1 mg/ml) or placebo during 6 weeks. The dose was 5 ml of oral solution. The main efficacy endpoint was the change from baseline of the nasal (4TSS) and global symptoms (5TSS) score at 4 and 6 weeks of treatment. Furthermore the assessment of children's quality of life at 4, 6 weeks by means of PRQLQ was also evaluated.

## Results

The subgroup analysed was a total of 266 randomized to rupatadine (n=131) or placebo (n=135). Table 1 summarizes the efficacy results:

**Table 1:** 

Mean score reduction	Placebo (n=135)	Rupatadine (n= 131)	P-value
4TSS at 4 weeks	-2.4 (1.9)	-3.1 (2.1)	P < 0.01
4TSS at 6 weeks	-2.6( 2.0)	-3.4 (2.1)	P < 0.01
5TSS at 4 weeks	-2.7 (2.4)	-3.7 (2.5)	P < 0.01
5TSS at 6 weeks	-2.9 ( 2.5)	-4.0 (2.6)	P < 0.01

PRQLQ overall score showed statistical significant differences between RUP and placebo at 4 weeks (p=0.01) and 6 weeks (p<0.05). Adverse events were scarce in both treatment groups throughout the study. Somnolence was reported with a very low incidence (1.4% RUP) and no serious adverse events were reported.

## Conclusion

Rupatadine oral solution (1mg/ml) was significantly more effective than placebo in improving nasal symptoms (4TSS) at 4 and 6 weeks. This is the first clinical evidence of a H1-receptor antagonist efficacy in children between 6-11 years over 25 kg with PER.

